# RNAi based transcriptome suggests genes potentially regulated by *HSF1* in the Pacific oyster *Crassostrea gigas* under thermal stress

**DOI:** 10.1186/s12864-019-6003-8

**Published:** 2019-08-08

**Authors:** Youli Liu, Li Li, Baoyu Huang, Wei Wang, Guofan Zhang

**Affiliations:** 1 Key Laboratory of Experimental Marine Biology, Institute of Oceanology, Chinese Academy of Sciences, Qingdao, 266071 China; 2 Laboratory for Marine Biology and Biotechnology, Qingdao National Laboratory for Marine Science and Technology, Qingdao, 266071 China; 3 University of Chinese Academy of Sciences, Beijing, 100039 China; 4 Laboratory for Marine Fisheries Science and Food Production Processes, Qingdao National Laboratory for Marine Science and Technology, Qingdao, 266071 China; 5 Center for Ocean Mega-Science, Chinese Academy of Sciences, Qingdao, 266071 China; 6 National and Local Joint Engineering Laboratory of Ecological Mariculture, Qingdao, 266071 China

**Keywords:** *Crassostrea gigas*, Heat shock, HSP, HSF1, Regulatory, RNA interference, Transcriptome, WGCNA

## Abstract

**Background:**

The Pacific oyster *Crassostrea gigas* is an important fishery resource that is sensitive to temperature fluctuations. Thus, it has evolved a protection mechanism against heat stress by increasing the expression of the gene coding for heat shock protein (HSP) 70 under elevated temperatures. In other animals, heat shock response is a transcriptional response driven by the heat shock transcription factor 1 (*HSF1*) and thermal stress can trigger *HSP70* expression to protect the organism via *HSF1*. However, the regulatory relationship between *HSF1* and *HSP* remains unclear in Pacific oyster. Therefore, in the present study, we examined the transcriptomic response of several to thermal stress following *HSF1* interference.

**Results:**

We identified 150 genes responsive to heat shock including seven *HSP* genes, six of which belonging to the group of 17 *HSP* genes enriched in response to heat shock, according to weighted gene co-expression network analysis (WGCNA). The other gene was enriched in the module correlated with *HSF1* interference. In addition, we found 48 and 47 genes that were upregulated and downregulated by *HSF1* in response to heat shock, respectively. In the upregulated genes, we identified one *HSP70* potentially regulated by *HSF1* in response to heat shock. Furthermore, based on differentially expressed genes and WGCNA analyses, we found that the hypoxia signaling pathway was enriched under heat shock conditions. Five genes were then selected to detect dynamic changes through time. The results suggested that gene expression was correlated with *HSF1* expression. The regulation of *HSP70* by *HSF1* was preliminarily confirmed by binding site predictions and by a dual luciferase assay.

**Conclusions:**

Our results revealed that the expression of *HSP70* and *HSP20* was initially triggered after 2 h of heat shock, and one of the *HSP70* genes was potentially regulated by *HSF1*. From these results, it is evident that not all heat-inducible genes were triggered simultaneously in response to heat shock stress. Overall, the results revealed a possible *HSF1–HSP* regulatory relationship in Pacific oyster, providing valuable information on the mechanisms of thermal tolerance in this commercially important oyster.

**Electronic supplementary material:**

The online version of this article (10.1186/s12864-019-6003-8) contains supplementary material, which is available to authorized users.

## Background

Temperature is an important environmental factor affecting the physiological metabolism [1], distribution [2], and growth [3] of organisms. Naturally distributed in the intertidal zone, Pacific oyster (*Crassostrea gigas*) are prone to stress and seasonal temperature fluctuations [4]. The species has become a valuable model for studying the adaptability of marine mollusks because its reference genome (GCA_000297895.1, GenBank) [4] is available. Due to its ability to adapt to a wide range of environmental conditions and high market value, *C. gigas* is an aquaculture resource in many countries. Temperature, an important abiotic factor, is related to *C. gigas* mass mortality during summer as individuals become more susceptible to infection by bacteria and viruses [5, 6], and high temperature hinders their development [7]. Tolerance to thermal stress requires compensation for the metabolic energy used through molecular regulatory processes.

The physiological effects of temperature fluctuations on the growth and metabolism of Pacific oyster have been well studied [8, 9]. Previous studies have focused on heat shock tolerance accumulation, detection of physiological indicators, and analysis of gene expression under different temperature treatments in terms of transcriptome [10–15] and proteome [16] changes. Many heat responsive genes were reported in these studies. Notably, *HSP* genes, which function as molecular chaperones, play an important role in heat shock response. Bivalves such as the Pacific oyster [4], the pearl oyster, *Pinctada fucata* [17], the golden mussel, *Limnoperna fortunei* [18], and the Yesso scallop, *Patinopecten yessoensis* [19] underwent massive *HSP70* gene family expansion compared with other animals [11], according to recent genomic and transcriptomic surveys. In addition, *HSP70* genes are particularly expanded and highly inducible during heat shock in oysters, suggesting that these genes play important roles in heat and other stress factors adaptation in oysters [20] and other bivalves [17–19, 21, 22].

Although heat responsive genes have been widely studied, their regulatory mechanisms are not fully understood. Heat shock transcription factor 1 (*HSF1*) can form trimers in response to thermal stress and it regulates the expression of heat shock response genes (especially *HSP* genes) by binding to heat shock elements (HSEs) in mammals [23, 24]. The isoforms of *HSF1* in Pacific oyster were described by Kawabe and Yokoyama [25], and studies on the functions of *HSF1* in other species provided insights into the molecular interactions that occur during heat shock. For example, the *HSF1–HSP* pathway has been described in the roundworm *Caenorhabditis elegans* [26, 27], in the fruit fly *Drosophila melanogaster* [28–30], and in mammals [31, 32]. Thus, we hypothesized that a similar pathway may exist in Pacific oyster.

RNA interference (RNAi) and sequencing are widely used to study the mechanisms of various life processes in *C. elegans* [27], *D. melanogaster* [33], and mammal cells [34]. Weighted gene co-expression network analysis (WGCNA), which can help to identify associations between genes and treatments, has also been widely used to identify hub genes of specific biological processes. In the present study, untreated and *HSF1*-interfered Pacific oyster individuals were subjected to a heat shock treatment at 35 °C for 2 h. In addition, oyster RNAi was used to identify genes potentially regulated by *HSF1*. Heat shock- responsive transcripts were revealed through transcriptome analysis the pathway in which *HSP70* is regulated by *HSF1* under heat shock conditions was illustrated. Using a dual-luciferase reporter assay and quantitative real-time PCR (qRT-PCR), we preliminarily validated the regulatory relationship and the expression patterns of enriched genes.

## Results

### RNA interference experiment

An initial experiment on heat shock and *HSF1* interference treatment times showed that *HSF1* was significantly highly expressed compared with the control after 2-h heat shock treatment. Therefore, 2 h was considered the adequate time for heat shock treatment in further experiments (Additional file 1: Figure S1) and 48 h was the adequate time to detect RNAi effects of *HSF1* (for gene expression levels see Additional file 1: Figure S2).

**Fig. 1:**
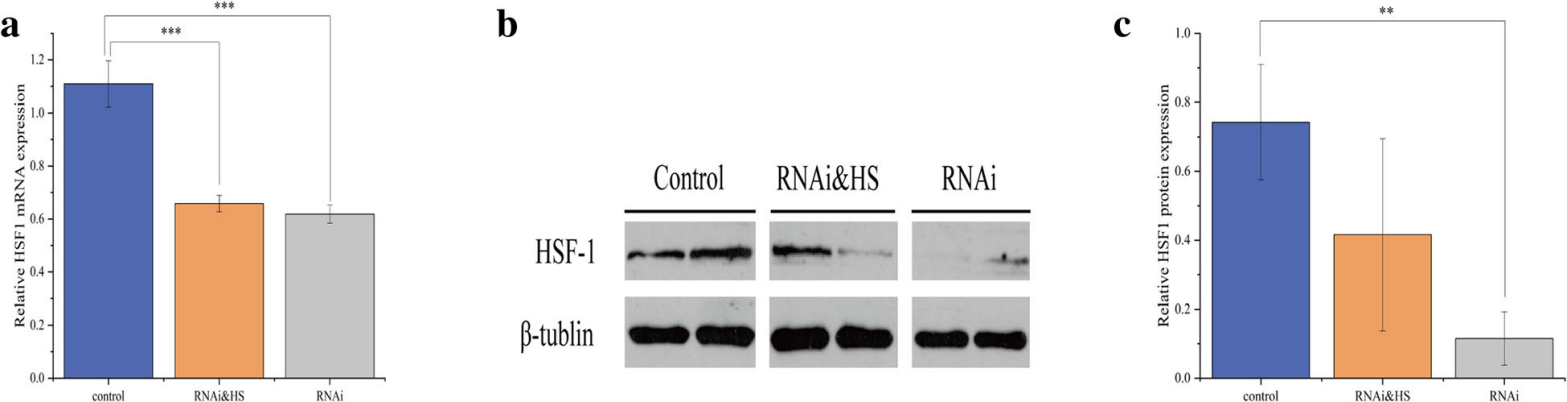
Effects of *HSF1* gene silencing on HSF1 expression in *C. gigas* gills. The control represents group 1, RNAi&HS represents group 4 (heat shock following RNA interference), RNAi represents group 3 (RNA interference) **a** *HSF1* mRNA expression detected by qRT-PCR (*n* = 15). **b** HSF1 protein content detected by western blotting (*n* = 2). Each band represents the protein content of an individual, with the upper band representing the content of the HSF1 protein and the lower band representing the content of β-tubulin. **c** Statistical analysis of HSF1 protein content detected by western blotting (*n* = 2). The error bar denotes the standard error of the mean; **, *p* < 0.05; *** *p* < 0.001

Results of the sequencing analysis for *HSF1* gene expression and protein content of the RNAi oysters with and without heat shock are shown in Fig. 1. qRT-PCR results showed no significant difference in *HSF1* mRNA expression between the RNAi group and the RNAi&HS group (*p* > 0.05), while both groups showed significantly lower expression of *HSF1* compared with that of the control group (both *p* < 0.001) (Fig. 1a). Western blotting was used to detect the expression of HSF1 protein in each group, with β-tubulin as the control (Fig. 1b), and protein content was measured using a gray scale (Fig. 1c). Protein levels were significantly different between the RNAi and the control groups (*p* < 0.05). Overall, these data indicated that our RNAi treatment was effective.

### Transcriptome data analysis

The mRNA of four groups subjected to different treatments [group 1: control; group 2: heat shock treatment (HS); group 3: RNAi; and group 4: RNAi&HS], and each consisting of five randomly assigned individuals with three biological replicates was sequenced in the Illumina HiSeq X Ten platform (Illumina, CA, USA). The high-quality 150 bp paired-end reads of 12 RNA-sequencing (RNA-seq) samples were mapped to the *C. gigas* genome (quality information for the 12 samples is shown in Additional file 2: Table S1). The high-quality reads of each sample were sequence-aligned with the *C. gigas* genome, and the efficiency of the alignment varied from 75.18 to 76.51%. Based on the alignment results, 7873 new genes were identified (new genes are listed in Additional file 3: Table S2), of which 6212 were functionally annotated in five databases (Additional file 4: Table S3). We normalized each condition (groups 1–3) to the control in order to determine fold changes in the relative abundance of mRNA. Differentially expressed genes (DEGs) [false discovery rate (FDR) ≤0.05, fold change (FC) ≥1.5] for groups 2, 3, and 4 compared with the control group are designated gene sets 1, 2, and 3, respectively, throughout this manuscript (Additional file 1: Figure S3). A complete list of the significant genes altered in response to each condition, after normalization to the control (150, 173, and 215 genes were identified for gene sets 1, 2, and 3, respectively), is provided in Additional file 5: Table S4 (FDR ≤0.05, FC ≥1.5). The DEGs were annotated using Gene Ontology (GO) and Kyoto Encyclopedia of Genes and Genomes (KEGG) databases (gene annotation is presented in Additional file 5: Table S4; annotation statistics are presented in Additional file 6: Table S5). To test whether high temperature and *HSF1* levels could affect the expressions of *C. gigas* housekeeping genes, we examined the changes in the expressions of elongation factor 1α (*EF-1α*), glyceraldehyde 3-phosphate dehydrogenase (*GADPH*), *β-actin*, *β-tubulin*, *18 s rRNA*, and *28 s rRNA* among treatments, but detected no significant differences (Additional file 1: Figure S4).

### Heat shock-responsive transcripts

According to GO classification, the 150 genes responsive to heat shock in gene set 1, i.e., genes that were differentially expressed between groups 1 and 2 (FDR ≤0.05, FC ≥1.5), were annotated in the following terms: “single-organism process” (GO:0044699, 37 genes) and “cellular process” (GO:0009987, 34 genes) for biological processes; “cell part” (GO:0044464, 25 genes) for cellular components; and “binding” (GO:0005488, 28 genes) and “catalytic activity” (GO:0003824, 21 genes) for molecular functions. Five *HSP70* and two *HSP20* genes were enriched compared with the control group (Table 1). Pathways that were significantly enriched (*p* < 0.05) under heat shock conditions are shown in Table 2, and they include apoptosis, protein processing in endoplasmic reticulum, ubiquitin-mediated proteolysis, the hypoxia-inducible factor (HIF)-1 signaling pathway, and the vascular endothelial growth factor (VEGF) signaling pathway.

**Table 1:** HSP in differentially expressed genes of heat shocked and control groups

ID	Annotation	FDR
CGI_10011376	HSP20	0.000535
CGI_10004164	HSP20	6.10E-07
CGI_10002387	HSP70	3.03E-86
CGI_10002823	HSP70	1.13E-10
CGI_10003417	HSP70	0.049901
CGI_10010647	HSP70	0.015578
CGI_10002594	HSP70	0.015987

**Table 2:** Enriched KEGG pathways in response to heat shock response compared with the control

KEGG_pathway	Ko_id	*P*-value
Apoptosis - multiple species	ko04215	0.000426
Spliceosome	ko03040	0.005705
Protein processing in endoplasmic reticulum	ko04141	0.006241
Ubiquitin mediated proteolysis	ko04120	0.016224
Endocytosis	ko04144	0.017012
Amoebiasis	ko05146	0.028273
Longevity regulating pathway - multiple species	ko04213	0.03291
Fanconi anemia pathway	ko03460	0.03451
Notch signaling pathway	ko04330	0.038701
HIF-1 signaling pathway	ko04066	0.042121
VEGF signaling pathway	ko04370	0.042121

*p* < 0.05 was significant

*KEGG* Kyoto Encyclopedia of Genes and Genomes

### DEGs potentially regulated by *HSF1* response to thermal stress

**Fig. 2:**
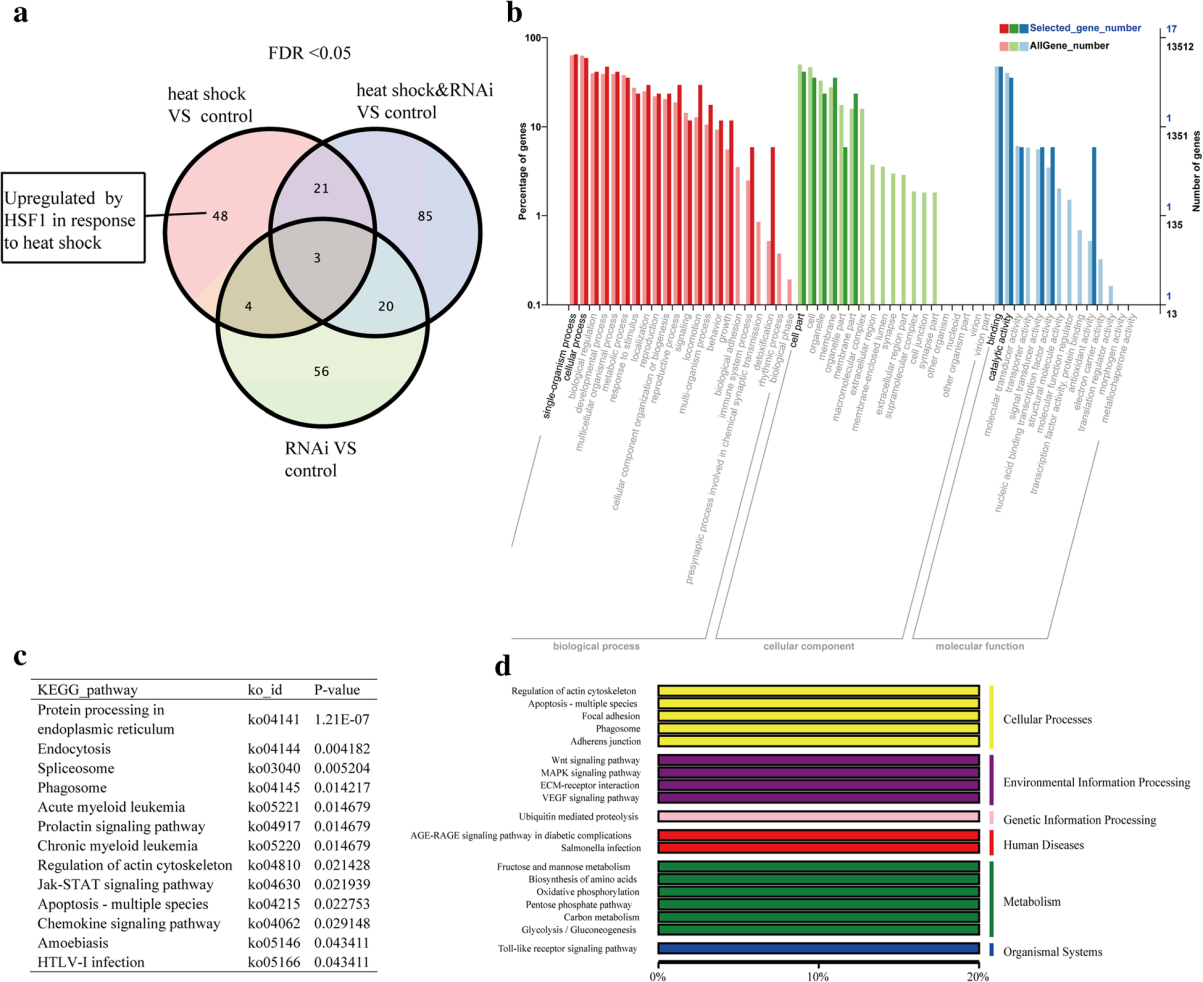
The DEGs upregulated by *HSF1* in response to heat shock (FDR < 0.05). **a** Venn diagram showing the overlap of three groups (groups 2–4) compared with group 1 (control). The pink area indicates genes that were upregulated by *HSF1* in response to heat shock. **b** Gene Ontology classification of the DEGs upregulated by *HSF1* in response to heat shock. The red bars represent biological processes; the green bars represent the cellular component; and the blue bars represent the molecular functions. **c** Kyoto Encyclopedia of Genes and Genomes (KEGG) pathways enriched for the *HSF1*-regulated genes, which were dependent on heat shock. **d** KEGG classification of the *HSF1*-regulated genes dependent on heat shock. Each row on the left side represents a pathway, and the collection to which the pathway belongs is displayed on the right side

The genes potentially regulated by *HSF1* in the early stage of heat shock were unique to gene set 1, after excluding those that overlapped with gene sets 2 and 3 (group details are presented in Additional file 1: Figure S3, S5). The 95 DEGs potentially regulated by *HSF1* included 48 upregulated genes (pink area in Fig. 2a) and the most abundant GO terms were the same as those for the heat shock treatment (Fig. 2b). The KEGG annotation of these DEGs (Fig. 2c, d) revealed that protein processing in endoplasmic reticulum, endocytosis, spliceosome, and apoptosis pathways were enriched. One *HSP70* gene (*CGI_10002387*), which was annotated in “posttranslational modification, protein turnover chaperones”, belonged to the DEGs unique to gene set 1.

**Fig. 3:**
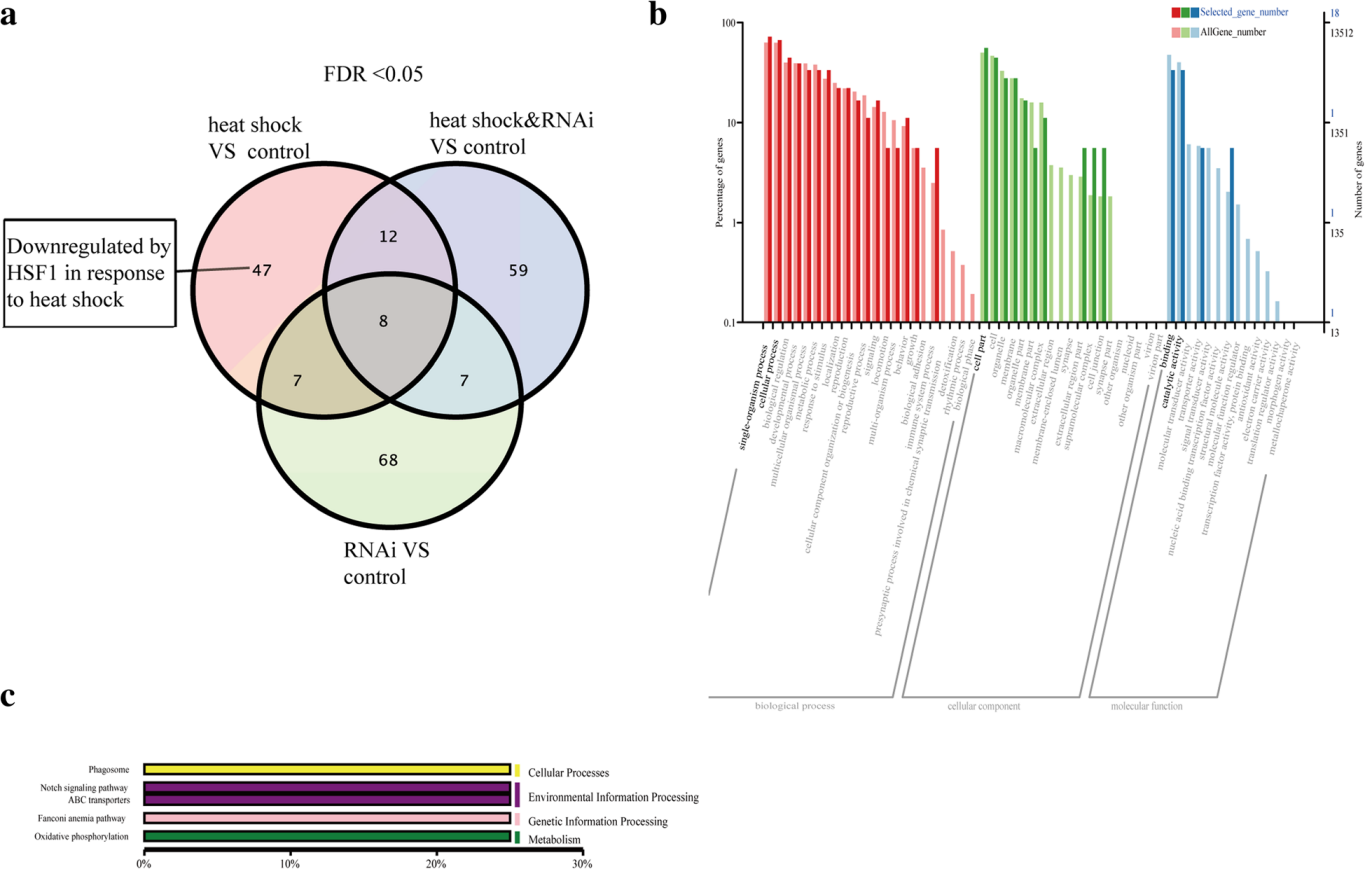
DEGs downregulated by HSF1 in response to heat shock (FDR < 0.05). **a** Venn diagram showing the overlapping groups (groups 2–4) compared with group 1 (control). The pink area indicates genes that were downregulated by *HSF1* in response to heat shock. **b** Gene Ontology classification of the DEGs downregulated by *HSF1* in response to heat shock. **c** Kyoto Encyclopedia of Genes and Genomes classification of the *HSF1* downregulated genes that were dependent on heat shock

Among the 95 DEGs unique to gene set 1, 47 were downregulated (pink area in Fig. 3a), and the most abundant GO terms were the same as those for the heat shock treatment (Fig. 3b). Five genes were annotated in KEGG, of which two were related to environmental information processing (Fig. 3c).

### Co-expression network

**Fig. 4:**
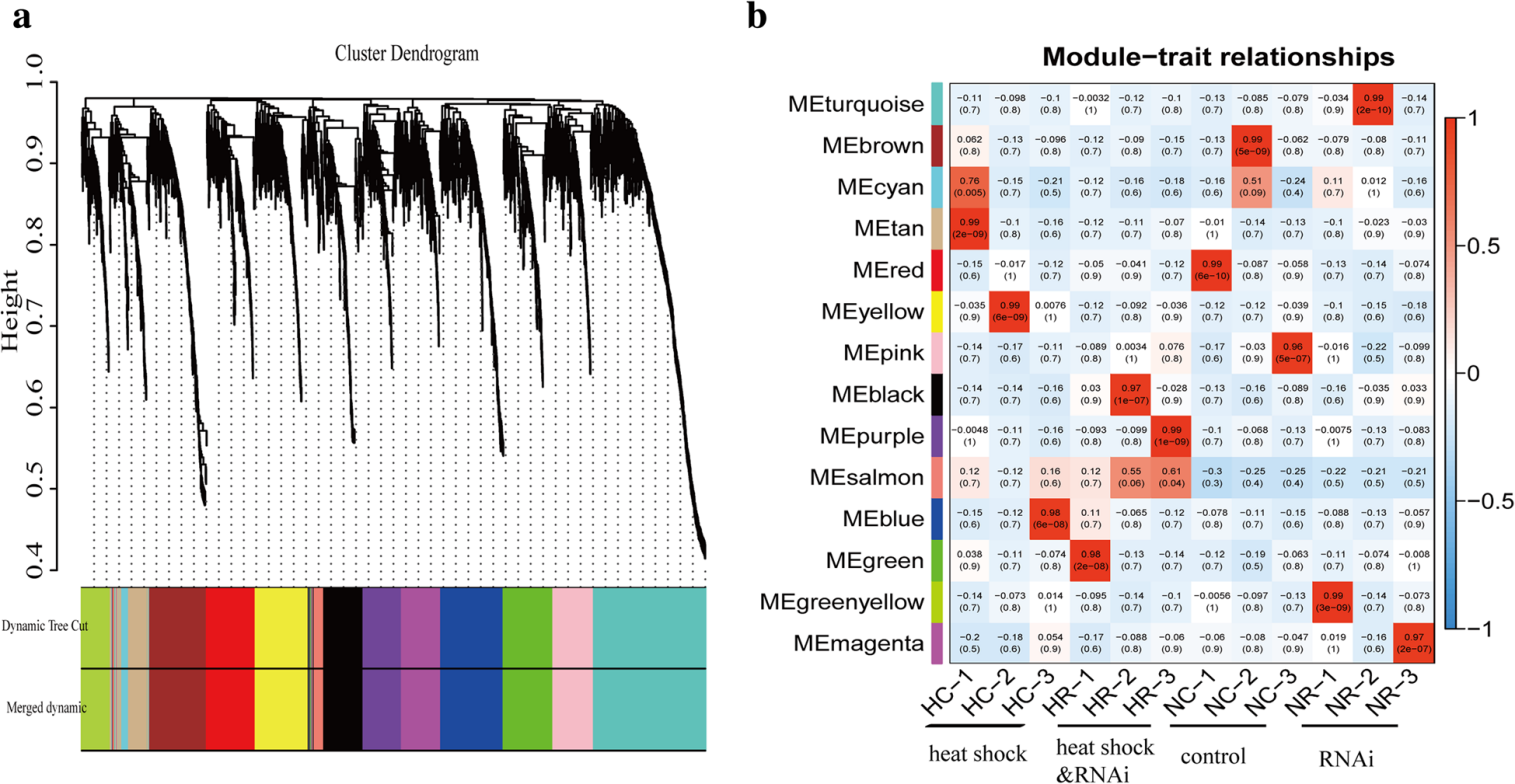
Weighted gene coexpression network (WGCNA) analysis of genes in all samples. **a** Hierarchical cluster tree representing the coexpression modules identified by WGCNA. Each leaf in the tree represents one gene. The major tree branches constitute 14 modules [fragments per kilo base of exon per million reads mapped (FPKM) ≥1, module similarity threshold ≥0.25], labeled with different colors. **b** Module–sample association. Each row corresponds to a module and it is labeled with a different color as in (**a**). Each column corresponds to a sample. The color of each cell at the row–column intersection indicates the correlation coefficient between the module and the sample. Each cell represents the correlation value between the module and each sample; the upper number is the correlation value, and the number underneath that is the *p*-value (significant at *p* < 0.05)

To find the genes responsive to HS and RNAi&HS treatments, we identified the co-expressed genes by applying WGCNA [35, 36] across all groups. The 2758 genes that fulfilled the stringent criteria (FPKM ≥1, module similarity threshold ≥0.25) belonged to 14 co-expression modules (14 different colors in Fig. 4) containing from 40 (cyan) to 499 (turquoise) genes (see details in Additional file 7: Table S6). The 14 distinct modules identified (Fig. 4a) are displayed as a single sample type to show the relationships between modules and sample (Fig. 4b). Although the three biological replicates of each condition belonged to different modules, these modules were correlated with each other (Additional file 1: Figure S6; the Eigengene adjacency heatmap shows that the black, purple, and salmon modules were highly correlated with each other, and that the blue module was positively correlated with the salmon module).

Under the heat shock treatment, 14 *HSP70* and three *HSP20* were enriched according to WGCNA (Additional file 8: Table S7). Three modules (tan, yellow, and blue) were strongly correlated with heat shock conditions. The tan module was highly correlated to the heat shock treatment sample (HC-1) and the genes were highly correlated with those of the tan module (*r* = 0.98, *p* < 0.05). The genes classified by GO analysis as “binding” in the molecular function and as “metabolic process” in the biological process categories were the most abundant. Pathway enrichment was observed for signaling transport, amino acid metabolism, carbohydrate metabolism, xenobiotics biodegradation and metabolism, and other catabolism processes. Notably, the *HIF-1* signaling pathway (Ko04066) was included in the environmental information process (*p* < 0.05). Both the yellow and blue modules were enriched for metabolism processing co-expression and were related to the heat shock treatment samples (HC-2 and HC-3, respectively). For the yellow module, lipid metabolism, ubiquitin-mediated proteolysis, and phagosome formation were the most abundant processes. Furthermore, the tricarboxylic acid (TCA) cycle and amino acid metabolism were the most abundant metabolic processes. The genes in the blue module were annotated in six kinds of processes. The most abundant were involved in genetic information processing such as RNA transport and protein processing.

Genes within group 4 were identified in the black, purple, green, and salmon modules, in which some heat shock protein related genes were included (Table 3). The salmon module was also associated with heat shock (Fig. 4b), and *HSP* genes were included in this module.

**Table 3:** HSP genes in salmon, black, purple, and green modules

Module name	Gene ID	Annotation
Salmon	CGI_10010647	HSP70 protein, MreB/Mbl protein, NAD-specific glutamate dehydrogenase
	CGI_10004164	HSP20/alpha crystallin family
	CGI_10011376	HSP20/alpha crystallin family
	CGI_10002594	HSP70 protein, MreB/Mbl protein, NAD-specific glutamate dehydrogenase
	CGI_10010646	HSP70 protein, MreB/Mbl protein, NAD-specific glutamate dehydrogenase
	CGI_10002823	HSP70 protein, MreB/Mbl protein
	CGI_10004165	HSP20/alpha crystallin family
Black	CGI_10004166	HSP20/alpha crystallin family
	Crassostrea_gigas_newGene_28734	HSP70 protein
Purple	CGI_10002387	HSP70 protein
Green	CGI_10018425	HSP70 protein

**Fig. 5:**
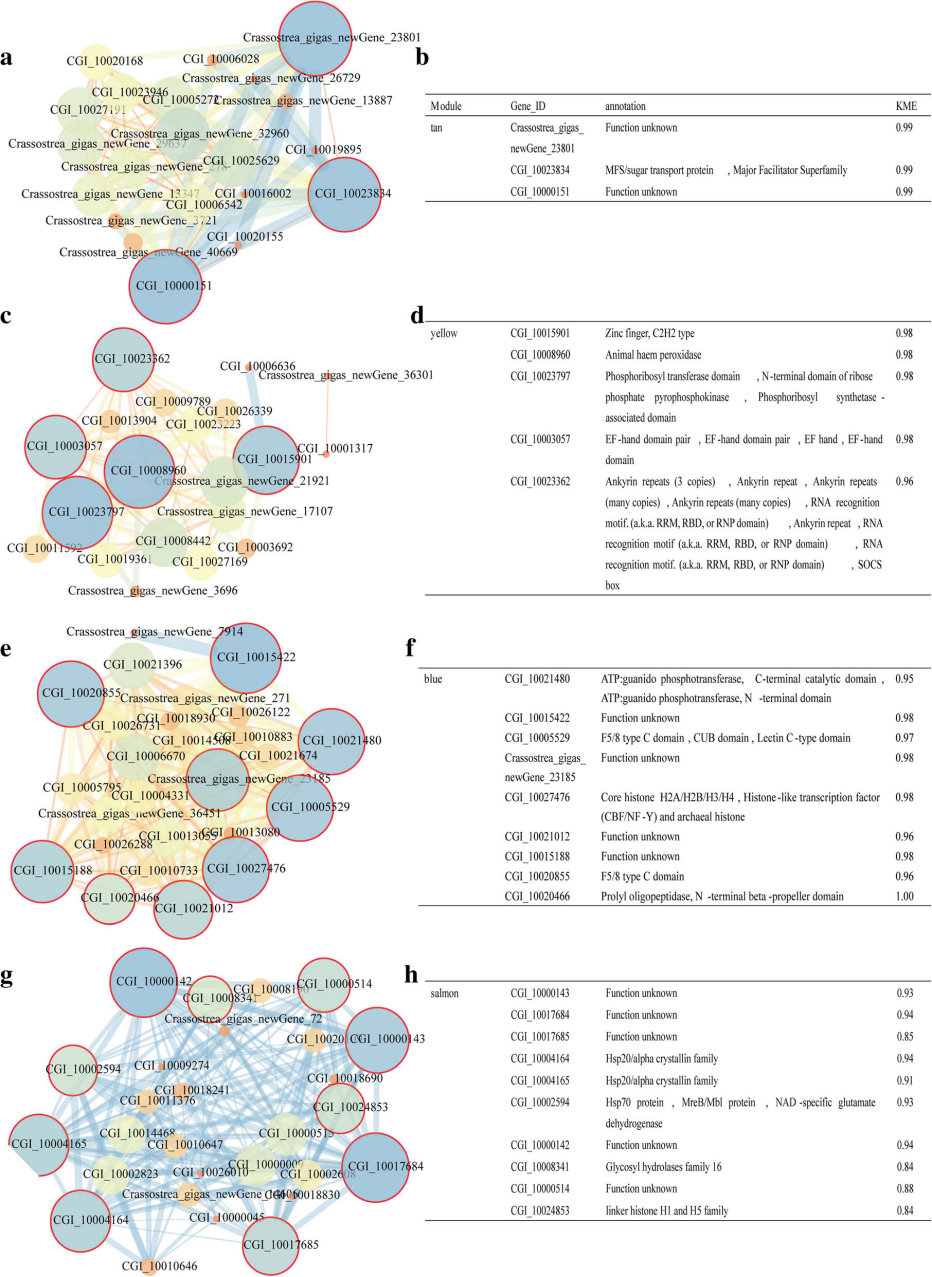
Correlation networks and genes with high K_ME_ values in the modules. **a**, **c**, **e**, and **g** represent tan, yellow, blue, and salmon modules, respectively. The larger the circle, the wider the lines between the genes and the higher the degree of connectivity between the gene and the module. **b**, **d**, **f**, and **h** represent the hub genes of the tan, yellow, blue, and salmon modules, respectively

Hub genes were selected by their high eigengene connectivity values (K_ME_ > 0.7) and showed the most connections in the network (i.e., the six genes with the highest K_ME_ among the four modules; modules related to HS and RNAi&HS are shown in Additional file 9: Table S8). The correlation network of the tan, yellow, blue, and salmon modules and their hub genes are shown in Fig. 5.

### Validation of the key responsive genes by qRT-PCR and dual luciferase assay

**Fig. 6:**
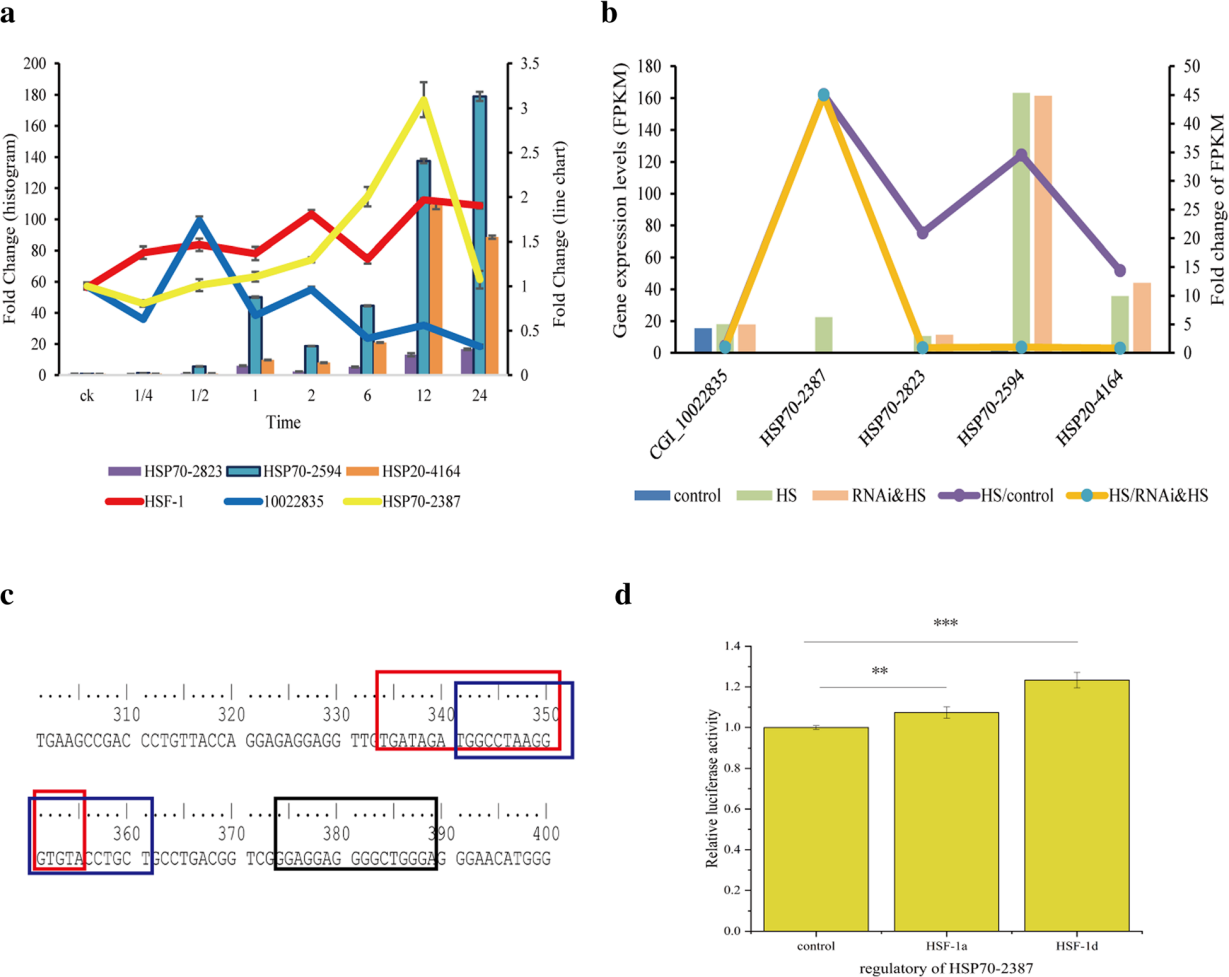
Quantification of enriched genes and validation of the regulatory relationship between *HSF1* and *HSP70–2387* genes. **a** Quantification of the expression of six genes at different sampling times under 35 °C heat stress treatment (*n* = 5). The X axis represents the sampling time from the start of the experiment (ck represents 0 h from the start, and sampling times were 0, 1/4, 1/2, 1. 2, 6, 12, and 24 h). The Y axis on the left represents fold change of the genes presented in the histogram while the right axis represents fold change of the genes presented in the line chart. The different colors represent the fold changes of each gene. **b** FPKM values and the fold change of enriched genes. Control represents group 1, HS represents group 2, and RNAi&HS represents group 4. The X axis represents different genes. The Y axis on the left represents FPKM of HS (heat shock) and control groups, while the right axis represents HS/control (FPKM of heat shock divided by FPKM of control) and HS/RNAi&HS (FPKM of heat shock divided by FPKM of RNAi&HS). **c** Sequencing of the *HSP70–2387* gene and the binding site of *HSF1* predicted by AnimalTFDB 3.0. The red, blue, and black frames represent the predicted heat shock elements. **d** Relative luciferase activity of the *HSF1* to the *HSP70–2387* genes. The control had no *HSF1* plasmid added to the system, and *HSF1a* and *HSF1d* represent the plasmids that can produce proteins containing the HSF1a and HSF1d isoforms of HSF1, respectively. The error bar denotes the standard error of the mean; **, *p* < 0.05; *** *p* < 0.001

Five genes, including three *HSP70* (*HSP70–2387*, *HSP70–2823*, and *HSP70–2594*), one *HSP20* (*HSP20–4164*), and one *HIF* signaling pathway-related (*CGI_10022835*) genes associated with heat shock, were selected for qRT-PCR analysis (materials treated at 35 °C for 0, 0.25, 0.5, 1, 2, 6, 12, and 24 h from the start of the experiment) and then compared with the RNA-seq data. The expression of the *HIF* signaling pathway-related gene changed under *HSF1* interference after 1 h of heat stress (Fig. 6a). The response of *HSP* genes was delayed but expression levels were higher than that of *HSF1* when continuously subjected to heat shock (Fig. 6a). The RNA-seq data showed that 2 h of heat shock significantly upregulated *HSP* genes and the expression level of each responsive gene under *HSF1* interference within 2 h of heat shock (Fig. 6b). The fold changes observed in these genes revealed that the mRNA expression of *HSP70–2387* changed the most when the organism was exposed to heat shock and had the highest interference level compared with that of other genes (Fig. 6b). This gene was one of the enriched genes unique to gene set 1 (after excluding gene sets 2 and 3), indicating that it might be regulated by *HSF1*. Thus, it was chosen to study the regulatory relationship between *HSF1* and *HSP70*. The predicted HSEs of *HSP70–2387* are listed in Fig. 6c, and the details of binding sequencing are listed in Fig. 6d. Dual luciferase assay results are shown in Fig. 6e. The transcription of the different isoforms of the *HSF1* gene was significantly different, but both isoforms (*HSF1a* and *HSF1d*) were positively activated.

## Discussion

Oysters are exposed to fluctuating temperatures that can lead to physiological stress owing to their intertidal habitat. As they can trigger the expression of stress-related genes for protection from heat stress, oysters might have regulatory capabilities [4, 8, 9, 16, 37]. Heat shock response genes were expressed under heat shock treatment at 35 °C for 2 h. Two *HSP20* and five *HSP70* genes were enriched among the 150 DEGs. The results of the WGCNA analysis suggested that the *HSP*s were highly associated with the heat shock treatment. Three *HSP20* and 14 *HSP70* genes were enriched in WGCNA analysis. Although some *HSP70* genes can be induced by a specific stress, there is little change in the expression level of heat shock cognates under stress or control treatments. The expression of four *HSP70* genes (*HSP70–10647, HSP70–2594, HSP70–10646,* and *HSP70–2823*) was 20 times higher under 35 °C than under the control treatment (Additional file 8: Table S7). Four of the five heat-induced genes reported by Zhang [4] were consistent with our results, but the expression of another heat-induced gene reported in Zhang’s study (*CGI_10003417*) was about five times higher under heat shock treatment than under the control treatment. The four heat-induced genes (Fig. 6a) exhibited increasing mRNA levels with heat shock treatment. Zhu [11] revealed the dynamic expression pattern of thermal stress-responsive candidate genes under heat shock (35 °C), and that the recovery stages trigger heat shock responses. Two of the four genes (*HSP70–2594* and *HSP70–2823*) were also reported by Zhu [11], who considered that these genes were highly induced in response to 35 °C heat shock. All these results suggest that the highly induced genes revealed in our study are credible.

The expression of *HSP70–2387* was over 45 times higher under heat shock conditions than under control conditions (Fig. 6b). Heat shock proteins are molecular chaperones that play important roles in protein folding and refolding, stabilization of denatured proteins, and protein transport under heat or other stresses [38–41]. In Pacific oyster, *HSP*s protect the organism from heat stress, and the high expression levels of *HSP70* genes indicate that they may contribute more to this protective function. These results suggest that the heat-induced genes (mostly *HSP70s*) may play important roles in response to heat shock conditions. Several reports have shown that *HSP*s can be triggered by thermal stress. In Pacific oyster, most individuals exposed to 37 °C for 1 h could survive a subsequent heat treatment (43–44 °C, 1 h), and the expression of a 69 kDa HSP was induced by heat shock, suggesting that these proteins play an important role in oyster survival under heat shock [10]. Heat shock proteins and genes involved in cellular homeostasis were the most highly expressed when *C. gigas* were kept at 25 °C for 24 days and then analyzed by RNA-seq [42]. The study also showed that gills were more sensitive than the mantle, and therefore we choose the gills for transcriptome analysis. Furthermore, Zhang [16] examined the proteome in gills under high temperature (38 °C, 1 h) and found that *HSP*s, including eight *HSP20* genes, three *HSP70* genes, and one small *HSP*, *HSP9/12*, were enriched. Our findings are consistent with these results, as *HSP20* and *HSP70* were initially triggered under thermal stress.

Notably, the *HIF*-signaling pathway and VEGF signaling pathway contained enriched DEGs in the KEGG annotation. One of the DEGs (*CGI_10022835*) was annotated as “angiopoietin 4”. Angiopoietins are a family of vascular growth factors that play a role in angiogenesis. It was reported that angiogenesis can be triggered during thermal stress in zebrafish [43]. Previous studies have revealed that *angiopoietin 4* and *VEGF* were expressed at higher levels during hypoxia in U87 glioblastoma cells [44]. Angiogenic genes, such as *VEGF*, are regulated by *HIF* during hypoxic conditions [45]. Baird [30] showed that *HIF1* could control heat shock pathways by regulating *HSF1* in adult flies (*D. melanogaster*). In addition, Kawabe and Yokoyama [25] reported the *HIF-HSF* pathway in Pacific oyster. According to a previous study, intermittent hypoxia occurs when Pacific oyster is exposed to heat shock [46], and the genes involved in aerobic energy metabolism are downregulated in response to the thermal stress [47]. A possible explanation for this might be that the *HIF1* signaling pathway is involved in the heat shock response process. In addition, some processes were enriched in the heat shock module, such as the TCA cycle, lipid metabolism, amino acid metabolism, and signal transduction. These results are supported by those of previous studies where metabolic processes were involved in heat stress responses in *Mytilus* [48], *C. gigas* [14, 15], and zebrafish [43]. Under heat shock conditions, these metabolic processes were regulated to compensate for the energy consumed to produce *HSPs* [48, 49].

Several reports have also shown that RNAi can be an efficient method for studying gene functions [50–52]. We chose three combinations of small interfering RNAs for RNAi to avoid activating the interferon system [53–56]. Overall, knockdown of the *HSF1* gene allowed examining gene functions when the oyster was under heat stress. By performing western blotting and qRT-PCR, we found that the levels of the HSF1 protein and mRNA were significantly different compared with the control, suggesting that these materials could be used for HSF1 function analysis. By analyzing the transcriptome data, we found which candidate genes could be regulated by *HSF1* under heat stress conditions. The results showed that the most abundant terms enriched in DEGs were also enriched under the heat shock treatment. Thus, *HSF1* might regulate the genes related to these functions. For the KEGG pathway analysis, most of the upregulated genes were annotated in: metabolism, cellular processes, and environmental information processing; for example, DNA repair and metabolism. In a previous study, *HSF1* was found to transactivate genes involved in heat shock as cytoprotective proteins [32]. The results of the present study indicate that the “antioxidant activity” process can be triggered as part of the *HSF1* regulation in response to heat shock. This finding is supported by the results of Madeira et al. [57] regarding two common fish species living in an environment commonly exposed to high temperatures.

Although *HSP70* genes are expanded in Pacific oyster, it remains unclear which genes are heat-inducible and how these genes are regulated. The expression level of *HSP70–2387* changed the most under heat shock conditions (Fig. 6b), and that of the other four genes (*HSP70–2823*, *HSP70–2594*, *HSP20–4164*, and *CGI_10022835*) were almost the same with or without *HSF1* interference under heat shock conditions, suggesting that *HSP70–2387* is more likely to be regulated by *HSF1* than the other four genes. Two isoforms of *HSF1* can be expressed under heat shock and be transferred to the nucleus where they form trimers and bind the HSE to regulate the expression of target genes. The dual luciferase assay has been used for examining regulatory relationships [58] and, in our study, it showed that *HSF1* (two isoforms) could activate *HSP70–2387* (*CGI_10002387*) in HEK293T cells. Increases in the expressions of the five *HSP* genes through time were detected by qRT-PCR, and previous studies have shown that *HSP* genes are involved in the heat shock process [4, 59]. Gene *CGI_10002387* was considered to be regulated by *HSF1* and it exhibited the same trend as *HSF1*, albeit a delay in the mRNA level, suggesting that the expression levels determined by the different methods are reliable. The *HSF–HSP* pathway has been reported in *C. elegans* [27] and in some mammals [31].

Although many genes could potentially be regulated by *HSF1*, only one *HSP70* gene was enriched in the unique gene set, therefore not providing the most informative result for understanding the HSF1-HSP regulatory relationship. According to our analyses, several reasons might account for this result. Firstly, the heat shock condition applied was not so extreme, and therefore, gene responses might not have been determined as significant by our statistical analyses. A study on *HSP70* overexpression in *D. melanogaster* larvae showed that although extra *HSP70* provides additional protection against the immediate damage from heat stress, abnormally high HSP70 concentrations can decrease growth, development, and survival to adulthood [60]. Two hours of heat shock at 35 °C might be a common condition for Pacific oyster, and thus, not all *HSP70*s would participate in protection owing to the high energy demands of the process. These results suggest that a trade-off between energy and cell protection might exist for response to heat shock stress. Although few genes were enriched, they provided insights into the initial response of the organism to heat shock conditions. Thus, the results of the present study are relevant for studying the molecular-level reaction and the regulatory mechanism underlying response to heat shock.

Secondly, the levels of HSF1 protein and mRNA might have been further reduced, but not abolished, by the RNAi treatment. The abundance of *HSF1* gene may be related to incomplete interference (~ 40%). Although 40% interference suppressed the target gene, the protein level also decreased; however, it should be adequate for the confirmation of gene knockdown effects. Therefore, although there are limitations to the present study, our results provide insights into gene function. With the development of technology, more advanced methods of RNAi can be used with model organisms, such as nematodes, to further study the gene function of mollusks.

## Conclusions

The present study was designed to determine the effects of heat shock stress and the regulatory mechanisms of *HSF1* when the Pacific oyster is exposed to elevated temperatures. The DEG and WGCNA analyses revealed that seven *HSP* genes and 17 *HSP* genes were enriched during heat shock, respectively. These included *HSP70* and *HSP20* genes only, suggesting that these genes were initially triggered under thermal stress. In addition, 48 genes were upregulated and 47 genes were downregulated by *HSF1*, and the regulation of one *HSP70* gene that was enriched in the upregulated gene set by *HSF1* was preliminarily validated. The dual luciferase assay allowed examining the regulatory relationship between *HSF1* and the enriched *HSP70* gene. In addition, the DEG and WGCNA analyses revealed that the *HIF* signaling pathway was associated with heat shock. These findings will be of interest for future studies aiming to understand the mechanisms underlying thermal tolerance in Pacific oyster.

## Methods

### Experimental materials

Pacific oyster (*Crassostrea gigas*) was cultured at Shentanggou (36°21′N, 120°41′E), Qingdao, Shandong province, China. After separating the oysters into monomers, 7-month-old growing oysters of intermediate size were selected for the experiment. These individuals were 58.6 ± 1.3 mm in height and 23.4 ± 1.6 g in weight (values are mean ± standard errors of the mean). During the acclimation period, individuals were fed spirulina powder, and the water temperature was maintained at an average of 12 ± 1 °C for 1 week (water was changed daily).

After the acclimation period, the group of individuals subject to this treatment only was used as the control group (group 1). Individuals that were then subjected to heat shock treatment at 35 °C for 2 h (group 2) were used for detecting heat shock responsive genes. The RNAi-treated group (group 3) and the group treated with RNAi and heat shock (group 4) were used for detecting genes regulated by *HSF1* in response to heat shock by comparing the genes expressed in these groups with genes expressed in the other two groups. A diagram of the experimental design is presented in Additional file 1: Figure S5. For the experimental design, we normalized each condition to the control (group 1). The difference between groups 2 and 4 is RNAi of HSF1, which means that without the HSF1 RNAi, gene set 1 should be similar to gene set 3. Therefore, the overlap of gene sets 1 and 3 means that the genes were regulated independently of *HSF1* gene in response to heat shock. For gene sets 1 and 2, the genes that overlap indicate that gene expression changed under either heat shock or HSF1 interference. The overlap of gene sets 2 and 3 indicates that the genes regulated by HSF1 were independent from heat shock. Overall, the genes unique to gene set 1, after excluding those that overlapped with gene sets 2 and 3, represented the one regulated by HSF1 in response to heat shock.

### RNAi and heat shock conditions

Small interference RNA (siRNA) was synthesized by GenePharma (Shanghai, China) and used for RNAi assays (details of the sequence are shown in Additional file 10: Table S9). After acclimation, individuals were anesthetized according to Suquet [61] (500 g MgCl_2_ + 5 L seawater + 5 L freshwater) and then randomly divided into four groups, the siRNA group (*n* = 42), the diethyl pyrocarbonate (DEPC) water group (*n* = 42), the negative control (NC) group (*n* = 15), and the control group (*n* = 42). The muscles of the individuals in the former three groups were injected with 100 μL of 10 μg/100 μL siRNA, DEPC water, or 10 μg/100 μL NC strands, and then the individuals in the four groups were equally divided into two sub-groups for the following treatments. One sub-group was placed into sea water at 35 ± 0.7 °C for 2 h, 46 h after the start of the experiment. At 48 h, their gills were sampled and frozen in liquid nitrogen until used for the qRT-PCR. The time and duration of the heat shock and selected siRNA were optimized in the experiment (see Additional file 1: Figure S1, S2). The control group was used to detect whether the RNAi decreased *HSF1* expression.

The qRT-PCR was performed in the ABI 7500 Fast Real-Time PCR System (Applied Biosystems, CA, USA) using the SYBR Green real-time PCR mix (Takara, Japan). The primers for gene expression detection were from Kawabe [25] (*HSF1* total) and Li [62] (*EF-1a*).

### Immunoblotting and protein quantification

Individuals in groups 1 (control), 3 (RNAi), and 4 (RNAi and heat shock) were used for *HSF1* protein detection (*n* = 2 per group). Their gills were firstly ground in liquid nitrogen, and proteins were extracted using a cell lysis buffer (Beyotime, Jiangsu, China). Proteins were quantified using the Bradford assay and incubated with 2× protein sodium dodecyl sulfate polyacrylamide gel electrophoresis buffer (Takara) at 100 °C for 3 min. Proteins were analyzed by western blotting using the anti-*HSF1* monoclonal antibody (unpublished data) and β-tubulin antibody (CWbiotech, China) as controls. Band intensity was quantified using ImageJ software (http://imagej.nih.gov/ij/).

### RNA preparation and sequencing

The gills were also used for total RNA extraction with an RNAprep Kit (Tiangen, China) according to the manufacturer’s instructions. The quality and quantity of RNA were assessed using 1.5% agarose gel electrophoresis and the NanoDrop 2000c UV-Vis Spectrophotometer (Thermo Fisher Scientific, MA, USA), respectively.

According to the analysis of *HSF1* expression, 15 individuals of each group (three replicates of five individuals) were selected for RNA-seq. RNA integrity was assessed using the RNA Nano 6000 Assay Kit and the Agilent Bioanalyzer 2100 system (Agilent Technologies, CA, USA). One microgram of RNA per sample was used for generating sequencing libraries with the NEBNext Ultra™ RNA Library Prep Kit for Illumina (NEB, MA, USA) following the manufacturer’s recommendations. Index codes were added to attribute sequences to each sample. Briefly, mRNA was purified from total RNA using poly-T oligo-attached magnetic beads. Fragmentation was carried out using divalent cations under elevated temperature in NEBNext First Strand Synthesis Reaction Buffer (5×). First strand complementary DNA (cDNA) was synthesized using a random hexamer primer and M-MuLV Reverse Transcriptase. Second strand cDNA synthesis was subsequently performed using DNA polymerase I and RNase H. The remaining overhangs were converted into blunt ends via exonuclease/polymerase activities. After adenylation of the 3′ ends of DNA fragments, NEBNext Adaptors with a hairpin loop structure were ligated to prepare for hybridization. To preferentially select cDNA fragments of 240 bp, the library fragments were purified with the AMPure XP system (Beckman Coulter, CA, USA). Then, 3 μl USER enzyme (NEB) was used with size-selected, adaptor-ligated cDNA at 37 °C for 15 min followed by 5 min at 95 °C. The PCR was performed with Phusion High-Fidelity DNA polymerase, universal PCR primers, and the index (X) primer. Finally, PCR products were purified (AMPure XP system) and library quality was assessed using the Agilent Bioanalyzer 2100 system (Agilent Technologies). Clustering of the index-coded samples was performed on a cBot Cluster Generation System using TruSeq PE Cluster Kit v4-cBot-HS kit (Illumina, CA, USA) according to the manufacturer’s instructions. After cluster generation, the library preparations were sequenced on an Illumina HiSeq X Ten platform and paired-end reads were generated.

### RNA-seq data analysis

The quality control of raw data in FASTQ file format was firstly processed through in-house scripts. The scripts were used to filter out adapter sequences, duplicated sequences, ambiguous reads (‘N’), and low-quality reads. At the same time, Q20, Q30, GC-content, and sequence duplication were calculated for the high-quality data. All downstream analyses were based on the high-quality data. Software HISAT2 [63] was used to map the reads on *C. gigas* genome. Based on the *C. gigas* genome sequence, mapped reads were spliced using StringTie [64] software and compared with the original genomic annotation information to find the transcripts which have never been annotated before in the reference genome (*C. gigas* genome) in order to identify new transcripts and new genes, thus complementing the original genome annotation information. New genes were screened by filtering out sequences encoding short peptide chains (< 50 amino acid residues) or containing only a single exon (in order to ensure the result of new gene discovering was reliable, we filtered out the sequence which contained only one exon according to the observation that most eukaryotic genes have introns [65]). Gene function was annotated based on the National Center for Biotechnology Information non-redundant, Protein family, Swiss-Prot, KEGG Orthology (KO), and GO databases. The fragments per kilo base of exon per million reads mapped (FPKM) [66] were used to estimate gene expression levels.

Differential gene expression analysis of selected groups was performed using DESeq2 [67], which provided statistical routines for determining differential expression in digital gene expression data using a model based on a negative binomial distribution. The resulting *p*-values were adjusted using the Benjamini and Hochberg approach for controlling FDR. Genes with adjusted *p*-values < 0.05 found by DESeq2 were considered differentially expressed. Venny 2.1 (http://bioinfogp.cnb.csic.es/tools/venny/index.html) was used to construct Venn diagrams with the significantly altered mRNAs for each condition (FDR < 0.05) compared to the control group. Gene ontology is an internationally standardized gene-function classification system consisting of terms that provide a global representation of gene functions using a controlled vocabulary. The GO enrichment analysis was performed using the GOseq R package [68]. Significantly enriched metabolic and signal transduction pathways containing DEGs were determined using KEGG pathway enrichment analysis and compared with the whole genome background. Software KOBAS [69] was used to test the statistical enrichment of DEGs in the KEGG pathways.

### Identification of coexpression modules

The R package WGCNA [36] was used to identify modules of highly correlated genes based on the FPKM data. Genes with low FPKM [FPKM < 1 for 12 samples (group 1: NC-1, NC-2, NC-3; group 2: HC-1, HC-2, HC-3; group 3: NR-1, NR-2, NR-3; group 4: HR-1, HR-2, HR-3)] or a module similarity threshold < 0.25 were filtered out. The resulting adjacency matrix was then converted to a topological overlap (TO) matrix with the TO similarity algorithm and genes were hierarchically clustered based on TO similarity. The Dynamic Tree Cut package (https://cran.r-project.org/web/packages/dynamicTreeCut/) was used to cut the hierarchal clustering tree, and modules were defined as the branches from the tree cutting. Modules with fewer than 30 genes were merged into their closest larger neighbor module. Each module was summarized by the first principal component of their scaled module expression profiles (referred to as module eigengene [ME]). Module eigengene-based connectivity (K_ME_) of a gene to a given module was calculated as Pearson correlation coefficient (PCC) between the expression levels (FPKMs) of the gene and the ME of the module using the K_ME_ algorithm. Finally, genes were reassigned using the module Merge Using K_ME_ Algorithm to ensure that each gene had the highest K_ME_ in its own assigned module.

### Visualization of hub genes

Genes with the highest degree of connectivity within a module are referred to as hub genes [36]. The top 150 connections (based on topological overlap) among the genes in each module ranked by K_ME_ were visualized by VisANT [70]. The GO term enrichment analysis of the WGCNA-identified coexpression modules was performed using a modified Fisher’s exact test in Blast2GO (https://www.blast2go.com/) software (FDR < 0.05). The GO annotations for coexpressed modules were obtained from the *C. gigas* genome (GenBank accession No., GCA_000297895.1). For each module, only protein-coding genes were subject to GO enrichment analysis, and the most specific biological processes were reported.

### Validation of RNA-seq data by qRT-PCR and dual luciferase reporter assay

The qRT-PCR was performed for individuals subjected to heat shock at 35 °C and sampled at 15 and 30 min, and at 1, 2, 6, 12, and 24 h. Five individuals were sampled each time. After sampling, total RNA was prepared using an RNAprep Pure Tissue Kit (Tiangen) following the manufacturer’s protocol. The quality and quantity of prepared RNA were assessed using 1.5% agarose gel electrophoresis and the NanoDrop 2000c UV-Vis Spectrophotometer (Thermo Fisher Scientific). Each RNA sample was reverse transcribed using a cDNA synthesis kit (Takara) for gene expression detection by qRT-PCR. The primer used for HSF-total was from a previous study [25]. We used the housekeeping gene *EF-1a* as a control gene. The primers used are listed in Additional file 11: Table S10. The qRT-PCR was performed as described in a previous section.

The dual-luciferase assay has been widely used in cell lines to rapidly and accurately determine the activity of a given promoter. The Dual-Glo® Luciferase Assay System (Promega, WI, USA) was used to estimate the effect of *HSF1* on the mRNA level of the target gene in HEK293T cells following the manufacturer’s instructions. The potential binding site, the area of the *HSP70* promoter where *HSF1* can bind, was predicted by AnimalTFDB 3.0, following Guo [71]. The region ~ 2 kb upstream of the *HSP70* gene (CGI_10002387) was cloned into the pGL3-basic luciferase reporter plasmid, and the *HSF1a* and *HSF1d* fragments were constructed to pCMV-N-Myc plasmids. Cells were co-transfected with varying amounts of pRL-TK, pGL3-basic-2387, pCMV-N-Myc-HSF1a/pCMV-N-Myc-HSF1d, and pCMV-N-Myc plasmids and reporter gene activity was measured using the Dual-Luciferase Reporter Assay System 24 h after transfection. Fluorescence was detected using the Varioskan Flash Multimode Reader (Thermo Fisher Scientific). According to the fluorescence value of each group, we estimated the regulatory activity of *HSF1*.

### Statistical analyses

Statistical analyses were performed using the software SPSS 16.0 (IBM, NY, USA). One-way analysis of variance (ANOVA) followed by Duncan’s test was used to evaluate the expression levels of *HSF1* and control genes. The results were significant at *p* < 0.05.

## Additional files

Additional file 1: **Figure S1** qRT-PCR of total HSF-1 gene during heat shock (35 °C for 24 h). **Figure S2.** Small RNA interference strands were selected and tested separately by measuring the expression level of *HSF-1*. **Figure S3.** Scheme for RNA-seq data normalization. **Figure S4.** Housekeeping genes share a similar expression profile between each RNA-seq treatment condition. **Figure S5.** Experimental scheme for RNA-seq. **Figure S6.** The eigengene adjacency heat map of 14 modules. (DOCX 413 kb)

Additional file 2: **Table S1.** Quality information of 12 sample data output statistics. (XLSX 10 kb)

Additional file 3: **Table S2.** New gene of *Crassostrea gigas* discovered after filtering (the parameters we applied with StringTie are: -p 6 --merge -F 0.1 -T 0.1 -i -eB -p 6). (XLSX 3207 kb)

Additional file 4: **Table S3.** The number of new genes that are annotated in each database. (XLSX 9 kb)

Additional file 5: **Table S4.** Significant genes altered in response to each condition after normalization to the control. (XLSX 115 kb)

Additional file 6: **Table S5** Number of differentially expressed genes (DEGs) in each database. (XLSX 9 kb)

Additional file 7: **Table S6.** Number of genes of the 14 modules. (XLSX 9 kb)

Additional file 8: **Table S7.** Heat shock proteins (HSPs) in the tan, yellow, blue, and salmon modules. (XLSX 12 kb)

Additional file 9: **Table S8.** Top six genes with the highest K_ME_ values in the tan, yellow, blue, green, black, purple, and salmon modules. (XLSX 10 kb)

Additional file 10: **Table S9.** SiRNA of the RNA interference assay. (XLSX 9 kb)

Additional file 11: **Table S10.** Primer for validation of the genes enriched by RNA sequencing. (XLSX 9 kb)

## Data Availability

The transcriptome datasets supporting the results of this study are available in the NCBI BioProject repository (Accession Number PRJNA516762, https://www.ncbi.nlm.nih.gov/bioproject/PRJNA516762) and in the SRA database (https://www.ncbi.nlm.nih.gov/sra) (Accession Number SRP181984).

## Abbreviations

DEGs: Differentially expressed genes
FDR: False discovery rate
FPKM: Fragments per kilo base of exon per million reads mapped
GO: Gene ontology
*HIF*: Hypoxia induced factor
HS: Heat shock
HSC: Heat shock cognate
HSEs: Heat shock element
*HSF1*: Heat shock transcription factor 1
*HSP*: Heat shock protein
KEGG: Kyoto Encyclopedia of Genes and Genomes
KO: KEGG orthology
ME: Module eigengene
NC: Negative control
qRT-PCR: Quantitative real-time polymerase chain reaction
RNAi: RNA interference
RNA-seq: RNA-sequencing
SEM: Standard error of means
siRNA: Small interference RNA
WGCNA: Weighted gene co-expression network analysis
