# Supplementary material for: RNAi based transcriptome suggests genes potentially regulated by HSF1 in the Pacific oyster Crassostrea gigas under thermal stress
Source: BMC Genomics. 2019 Aug 8;20:639. doi: 10.1186/s12864-019-6003-8 (PMC6688261; doi:10.1186/s12864-019-6003-8)
Supplement: Supplementary file 1 — Figure S1 qRT-PCR of total HSF-1 gene during heat shock (35 °C for 24 h). Figure S2. Small RNA interference strands were selected and tested separately by measuring the expression level of HSF-1. Figure S3. Scheme for RNA-seq data normalization. Figure S4. Housekeeping genes share a similar expression profile between each RNA-seq treatment condition. Figure S5. Experimental scheme for RNA-seq. Figure S6. The eigengene adjacency heat map of 14 modules. (DOCX 413 kb) [file 12864_2019_6003_MOESM1_ESM.docx]

**Figure S1.** qRT-PCR of total *HSF-1* gene during heat shock (35°C for 24 h). Oysters were subjected to heat shock (35°C) and sampled for 24 h (at 0, 0.25, 0.5, 1, 1.5, 2, 3, 6, 12, 24 hour from the start). We use one-way ANOVA followed by a post hoc multiple comparison (Duncan) was conducted to test the significance of the expression levels through times (p<0.05).


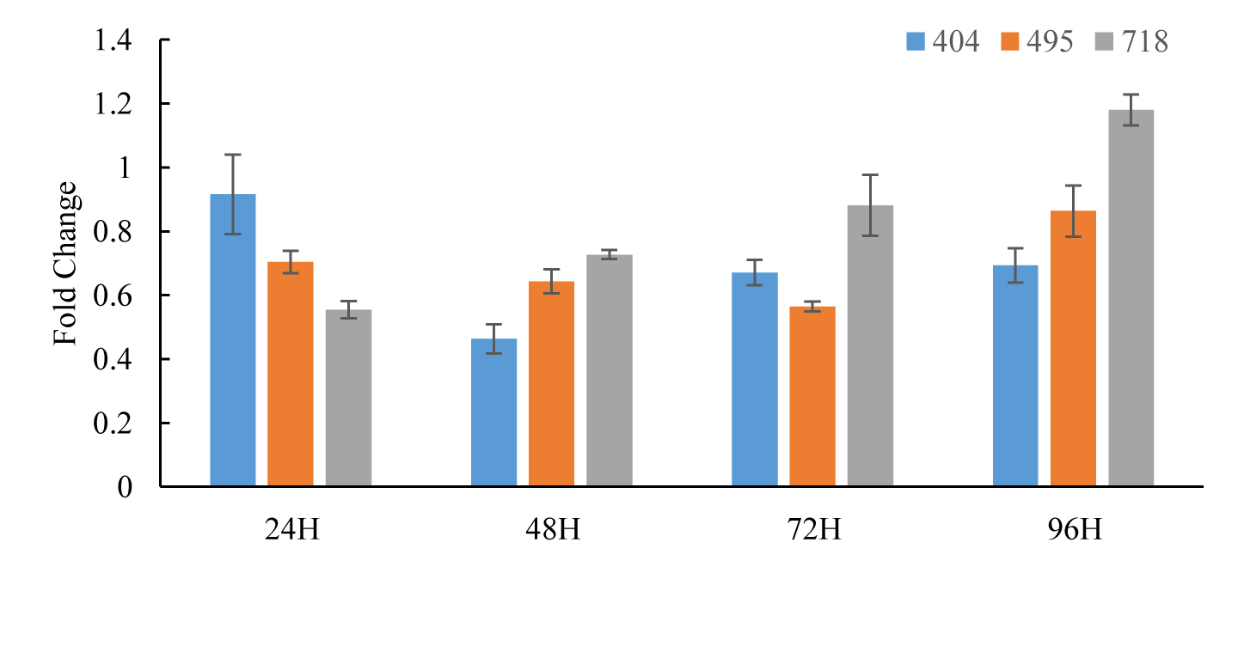


**Figure S2.** Small RNA interference strands were selected and tested separately by measuring the expression level of *HSF-1*. The qRT-PCR were taken using the cDNA of the *C.gigas* gill which were injected with the siRNA after anesthetized with the MgCl_2_. The horizontal axis represents time of the 24, 48, 72, 96 hour from the experiment start and the legend shows the number of each small RNA. “404”, “495” and “718” represent the name of the small RNA interference strands.


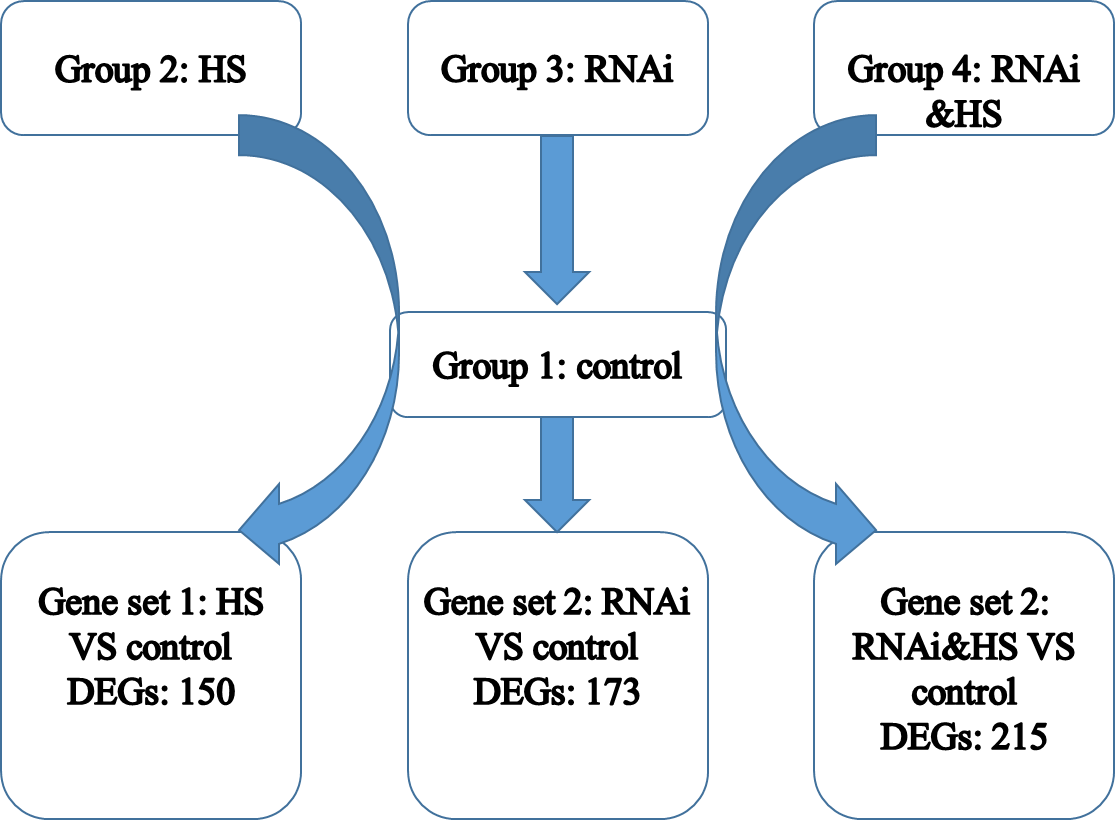


**Figure S3.** Scheme for RNA-seq data normalization. Each treatment condition was compared relative to the control group in order to determine fold changes in gene expression in mRNA level. “Gene Cluster1” represent the Different expressed genes (DEG) of Group 2 compared with Group 1; “Gene Cluster 2” represent the DEGs of Group 3 and Group 1; “Gene Cluster 3” represent the DEGs of Group 4 compared with Group 1. Different expressed genes (DEGs) represent the genes whose false discovery rate (FDR) ≤0.05 and fold change (FC) ≥1.5.





**Figure S4.** Housekeeping genes share a similar expression profile between each RNA-seq treatment condition. The fold change of treatment shows that no significant expression level occur between heat shock, RNAi, RNAi& heat shock and control group.

HSF-1 RNAi


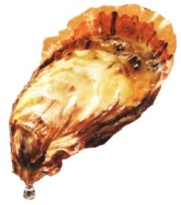


control


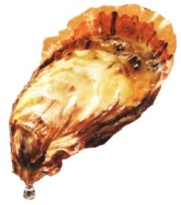


-Heat Shock

Illumina sequencing

+Heat Shock(2h 35℃)

**Figure S5.** Experimental scheme for RNA-seq. The oyster were generated from Shentanggou farm of Qingdao, China. RNA sample from gill of wild type oyster were generated, in three biological replicates (which including five individuals), under the four conditions: wild type referring to the oyster without any treat except accumulation for an week in the laboratory environment as control of other treat group; HSF-1 RNAi referring the oyster with HSF-1 siRNA treatment; “-Heat Shock” indicates that oysters stay at acclimation temperature (12°C), While “+ Heat Shock” indicates treatment with a 2 hour 35°C heat shock after 46 h HSF1 interference.


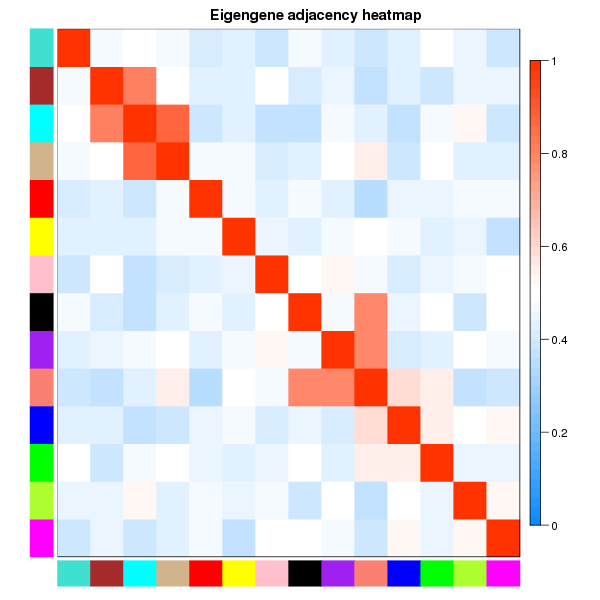


**Figure S6.** The eigengene adjacency heat map of 14 modules. Color of each cell represents the correlation value of the modules.
